# Immortality Reconsidered: Clinical Challenges at the Frontier of Plastic Surgery

**DOI:** 10.3390/jcm14227973

**Published:** 2025-11-10

**Authors:** Helen Xun, Audrey K. Mustoe, Maria J. Escobar, Zion Chan, Andrea Moreira, Sarvam TerKonda, Lynn Jeffers, Bernard T. Lee

**Affiliations:** 1 Division of Plastic and Reconstructive Surgery, Beth Israel Deaconess Medical Center, Harvard Medical School, Boston, MA 02115, USA; hxun@bidmc.harvard.edu (H.X.); amustoe@bidmc.harvard.edu (A.K.M.); escobardomingomj@upmc.edu (M.J.E.); 2 Division of Plastic and Reconstructive Surgery, Shanghai Fourth People’s Hospital, Tongji University, Shanghai 200070, China; 2405893@tongji.edu.cn; 3 Department of Plastic Surgery, University of Pittsburgh, Pittsburgh, PA 15260, USA; moreiraaa@upmc.edu; 4 Division of Plastic and Reconstructive Surgery, Mayo Clinic, Jacksonville, FL 32224, USA; terkonda.sarvam@mayo.edu; 5 St. John’s Regional Medical Center, Oxnard, CA 93030, USA; lynnjeffersasps@gmail.com

**Keywords:** immortality, healthspan, anti-aging, plastic surgery, aging, regenerative medicine, rejuvenation

## Abstract

**Background/Objectives:** Immortality and anti-aging research is accelerating, with implications across medicine. This narrative review explores the biological principles, translational innovations, and ethical considerations at the intersection of aging and plastic surgery, reframed for a broad clinical audience. **Methods:** A narrative review of the literature from PubMed, clinical trials, and translational studies was conducted, with emphasis on regenerative medicine, stem cells, tissue engineering, gene editing, and longevity pharmacologics within the field of plastic and reconstructive surgery. **Results:** Key themes include (1) the biology of aging and epigenetic reprogramming, (2) esthetic and regenerative innovations with broader clinical significance, (3) emerging genetic and pharmacologic longevity strategies, (4) ethical and regulatory challenges, and (5) future directions such as nanotechnology, artificial intelligence, and digital immortality. **Conclusions:** Immortality remains an aspirational frontier, but innovations in regenerative science and longevity research offer opportunities for improving healthspans. Medicine as a whole must balance innovation with ethics, equity, and safety in translating these discoveries to patient care.

## 1. Understanding Aging: A Multifaceted Process

Aging is a multifactorial biological process influenced by genetic, environmental, and lifestyle factors. Plastic surgery, traditionally focused on form and function, has become a translational partner in regenerative medicine. The field of plastic surgery does not focus on a singular organ system but on the philosophy and specific techniques of harmonizing form and function for lifesaving or life-changing procedures. These range broadly from cancer reconstruction to limb salvage, congenital craniofacial abnormality reconstruction, craniomaxillofacial trauma, or hand surgery. As advocates of patient quality-of-life, plastic surgeons are keenly aware of the evolving landscape of anti-aging, healthspan interventions, and the tantalizing prospects of immortality [1]. This narrative review on biological immortality delves into the scientific principles, research findings, and emerging trends surrounding biological immortality and anti-aging in plastic surgery as contributions to broader medical understanding of aging, healthspan, and longevity. In this narrative review, we identified original, peer-reviewed publications on PubMed related to immortality, anti-aging, rejuvenation within the field of plastic and reconstructive surgery or those published in plastic, reconstructive, or esthetic surgery journals.

Typically, chronological age correlates with age-related-diseases (ARDs) and increased rates of mortality [2]. However, in the concept of biological immortality, physical aging is stopped or reversed, separating biological age from chronological age. Biological aging is a complex, multifaceted process influenced by genetic, environmental, and lifestyle factors. There are many talented species in nature that have developed mechanisms to evade aging (Table 1) and have helped further elucidate the mechanisms of aging [3]. The cellular succession of biological aging is hypothesized to be an accumulation of DNA damage, mitochondrial dysfunction and free radicals, and telomere shortening. Ultimately these factors lead to the loss of biological information transmission and preservation, resulting in cellular apoptosis, stem-cell depletion, and cellular senescence [4].

Conventionally, the somatic mutation theory of aging suggests that DNA mutation accumulation is the intermediary. In recent years, the newly emerging Information Theory of Aging proposes that the primary mechanism of aging is disruptions in the epigenomes. For example, dual-function epigenetic and chromatin factors (such as sirtuins, DNA methyltransferase, or polycomb repressive complex 2) maintain genes and repair DNA damage sites. However, following DNA damage repair, these factors may not always revert to their original loci and restore previous patterns of gene expression [11]. Ultimately, these repeated cycles of DNA damage repairs can diminish epigenomic information, resulting in epimutations and loss of youthful cellular characteristics [11]. This theory aligns with the concept of developmental origins of health and disease, which postulates that physiologic responses to stress leave an epigenetic footprint that is beneficial in early life but may be harmful later in life, thus accelerating aging [12].

Emerging evidence has also demonstrated that cells may contain a “backup copy” of the epigenome that allows aging to be reversible or reset. In 2006, Shinya Yamanaka and his team identified the Yamanaka nuclear transcription factors: OCT4, SOX2, KLF4, and c-MYC (collectively known as “OSKM”). Expression of the OSKM transcription factors erases somatic cell identities, transforming them into pluripotent stem cells with an epigenetic age of zero [13,14]. Ex vivo studies of OSK/OSKM overexpression restored youthful gene expression patterns in fibroblasts, adipocytes, mesenchymal stem cells, and organs such as the kidney, liver, heart, brain, pancreas, and muscles [11,15]. In vivo studies involving pulsatile expression of OSK/OSKM in mice extended their lifespans^9^ and improved visual function in glaucomatous mice, without increasing the risk of tumorigenesis.

The importance of epigenetics in aging is further influenced by environmental epigenetics, which refers to how lifestyle choices and environmental exposures trigger epigenetic changes resulting in aging and disease [11,16]. The landmark Danish Twin Study in 1996 initially established that lifespan is dictated 80% by environment and 20% by genes [16]. However, more recent research suggests that lifestyle and environment may account for as much as 90% of the lifespan [17]. Biological aging is also closely linked to environmental toxicity [18], such as exposure to pesticides, metals, and air pollution [19,20,21,22]. Studies looking at social environmental factors, such as the practice of redlining communities in the United States, have demonstrated a correlation between low-income communities of color with epigenetic predictors of all-cause mortality [23], shortening of telomeres [24], and accelerated epigenetic age [25].

## 2. Plastic Surgery and Anti-Aging: Bridging Science and Esthetics in Current Practice

Plastic surgeons are at the forefront of healthspan [26], a longevity metric defined as the duration of healthy or disease-free life with maintained quality-of-life [27]. Currently available interventions focus on rejuvenation—restoration rather than prevention of aging. Facial rejuvenation procedures often performed by plastic surgeons, such as facelifts, brow lifts, eyelid surgery, and polydioxanone threads, aim to address age-related changes in facial contours and skin laxity [28]. In addition, injectable hyaluronic acid fillers and botulinum-toxin injections offer temporary, minimally invasive solutions for rhytid reduction and restoring volume. Other minimally invasive modalities commonly employed by plastic surgeons include microneedling, laser resurfacing, chemical peels and non-invasive skin tightening procedures, which enhance skin firmness and elasticity, exfoliate and remove damaged layers of skin, and stimulate cell turnover, collagen, and elastin synthesis and the release of growth factors [29,30,31,32].

While the aforementioned interventions may mitigate social morbidities of aging, emerging new treatments target genetic causes of aging, allowing for both rejuvenation and regeneration. Treatments such as poly-L lactic acid (PLLA) and polydeoxyribonucleotides (PDRN) reportedly address the senescence of dermal fibroblasts to promote tissue regeneration. PLLA is a biodegradable polymer microsphere that is injected into target areas, triggering foreign body responses that activate fibroblasts and promote neocollagenesis, resulting in long-lasting volume and improved skin texture and elasticity [28,33]. When combined with radiofrequency microneedling, PLLA results in increased dermal thickness and elasticity, with no lipolysis [34]. PDRN contains DNA fragments derived from salmon sperm, and possesses anti-inflammatory, angiogenic, and tissue-repairing and regeneration properties [35]. It has also shown promise in endosinus bone regeneration [36] as well as collagen regeneration [37]. Facial fillers containing PDRN not only provide volume support but also promote tissue regeneration by activating adenosine receptors and stimulating growth factor production [38,39,40]. Recent in vitro adaptations of PDRN into liposomal formulations show potential for improved incorporation into cosmetic skin formulations for skin permeability [41]. Additionally, PDRN may have properties that promote restoration of hair loss and treatment of hyperpigmentation, although further research is needed [42,43].

Photobiomodulation has recently emerged as a promising intervention to enhance cellular function and tissue repair. IR therapy—also known as low-level laser/light therapy (LLLT) or photobiomodulation—has demonstrated profound effects on cellular energy production, inflammation reduction, and tissue regeneration. Mechanistically, IR stimulates mitochondrial components such as cytochrome c oxidase, boosting ATP synthesis and promoting healing across varied tissues, including neural and dermal systems. Biological and medical reviews underscore IR’s benefits in neural stimulation, wound healing, and even oncologic contexts [44]. Clinical studies and in vivo models show that near-infrared LED (nNIR) therapy enhances skin thickness, accelerates collagen synthesis, and reduces collagenase expression—benefits supported both at the cellular level and in animal models of photoaging. Notably, nNIR also stimulates hair follicle growth. Photobiomodulation using NIR light has been shown to rejuvenate aged mesenchymal stem cells by restoring their mitochondrial function—indicating a potential role in enhancing regenerative capacity for tissue engineering and repair. While promising, broader clinical validation, optimized dosing protocols, and long-term safety studies are still needed to fully establish IR therapy’s role in regenerative medicine.

Platelet-rich plasma (PRP) therapy and regenerative fat grafting have emerged as pivotal modalities in the enhancement of tissue regeneration, thereby constituting areas of significant interest in anti-aging research. PRP involves the extraction and concentration of platelets and growth factors from the patient’s own blood, which are then introduced into the targeted areas to stimulate cell proliferation, angiogenesis, and collagen production. Recent reports have demonstrated the potential for PRP to restore hair [45], facilitate wound healing, enhance healing after facelift procedures [46], and improve fat or bone graft take, regeneration, and early vascularization [47,48,49]. Similarly, fat grafting and tissue transfer techniques also have regenerative properties, harnessing the benefits of stem cell therapies [47,50,51]. However, these techniques are distinct from stem cell therapy (such as stromal vascular fraction isolation, which contains higher concentrations of stem cells) [52]. These innovative approaches harness the body’s innate regenerative capacity to enhance wound healing and repair tissue and organ damage associated with chronological aging.

Can this regenerative capacity be borrowed? In mice, heterochronic parabiosis studies (surgically connecting a young and old mouse’s circulatory system) and heterochronic blood exchange studies (serum exchange between mice), showed age reversal for the older mouse due to the potential regenerative factors in blood [53]. These serum sharing studies found improved proliferation and regeneration in aged skeletal muscle stem cells, increased hepatocyte proliferation [54], neural progenitor cell proliferation, enhanced neurogenesis, and the prevention of cognitive decline [55]. The momentum carried quickly into human trials but continues to be under rigorous investigation and development. For example, a 2019 randomized controlled trial involving young plasma transfusions in nine patients with Alzheimer’s disease showed that the treatment was feasible and safe but did not demonstrate any efficacy [56]. A 2025 observational study examined heterochronic blood targets in the cerebrospinal fluid of 35 patients with Alzheimer’s disease, revealing that higher levels of rejuvenation proteins predicted better cognitive and functional outcomes [57]. However, further research must be conducted to better elucidate how best to harness youthful blood factors for their anti-aging effects.

There is a notable and growing trend globally in the establishment of longevity and healthy-aging clinics that offer a mix of conventional, integrative, and homeopathic therapies [58]. These facilities span a broad spectrum—from esthetic-focused medical spas to holistic wellness centers and integrative, evidence-based functional medicine practices. Many offer a mix of services, including advanced diagnostics, hormone optimization, IV nutrient infusions, acupuncture, lymphatic therapy, and rejuvenation procedures. This expansion is fueled by increasing public interest in geroscience and healthspan interventions. Notably, academic institutions and publicly funded hospitals are beginning to establish physician-led healthy longevity clinics, integrating multidisciplinary care models with research and standardizing protocols for early detection and intervention in age-related decline [59]. While these clinics have the potential to improve public wellness, quality of life, and life extension, there remains a regulatory gap. No comprehensive federal framework currently governs these longevity-focused clinics—standard medical practice laws and FDA guidelines apply, but tailored oversight for longevity medicine is absent. To address this, professional societies, such as Healthy Longevity Medicine Society, have emerged to promote professional and safety standards and develop clinical best-practice guidelines [60].

## 3. From Theory to Practice: Future Needs in Anti-Aging and Regenerative Medicine

While the research on longevity is expanding, there is a significant gap between anti-aging and amortality (extended lifespan). This gap can be approached using a foundational principle in plastic surgery: the craft of coaxing tissue into realizing new potentials beyond their genetic predispositions. Consequently, plastic surgeons and scientists contribute unique insights at the forefront of regenerative medicine and modulation of aging [26], including techniques such as stem cell therapies, tissue engineering, and gene editing (Table 2).

Plastic surgery research spans a broad spectrum of regenerative and rejuvenative sciences, as seen in Table 2, organized by the major plastic surgery subspecialties. Recent highlights in plastic surgery basic science research are summarized below. Stem cell therapies involve the cultivation, transformation, or transplantation of stem cells, which possess the remarkable ability to differentiate into various cell types and promote tissue regeneration through paracrine signaling [61,62,63], immunomodulation, and direct cell replacement. Animal studies in plastic surgery include the prevention of radiation disorders [64], enhancement of soft-tissue graft survival and neovascularization [65,66], improvement in wound healing and remodeling [67,68,69,70,71], treatment of lymphedema [72] or alopecia [73,74], and regeneration of peripheral nerve [74], bone [75], or muscle [76,77,78,79,80]. Similarly, tissue engineering exemplifies biological plasticity by creating functional constructs of scaffolds, cells, and biologics to treat or enhance tissues and organs [81]. Proof-of-concept studies in humans have included breast reconstruction with a mesh scaffold and fat graft [82] and arteriovenous loops with bone marrow aspirate for bone defects [83]. Current directions in plastic surgery tissue engineering research include vascularized soft tissue [84,85,86] and flaps [87], breast, calvarium and bone [88,89], skin [90,91], auricular cartilage and constructs [92,93,94,95,96], trachea [97,98], tendon [99], and nasal cartilage [100]. Furthermore, advancements in gene editing technologies offer unprecedented opportunities for the targeted manipulation of aging-related genes and pathways and the reprogramming of cellular function, as well as for the investigation of these pathways and therapeutic regeneration [101]. Clustered regularly interspaced short palindromic repeats (CRISPR)/CRISPR-associated protein 9 (Cas9) genome editing investigations in plastic surgery include enhancing cellular therapy and tissue engineering, wound healing by targeting genes encoding growth factors and cytokines, targeting congenital craniofacial syndromes [102], and repairing or regenerating bone, cartilage, nerve, and muscle. Mouse models have demonstrated the efficacy of utilizing CRISPR to effectively excise genetic mutations known to cause human microtia [103]. However, additional investigations and ethical considerations surrounding stem cell therapies, tissue engineering, and gene engineering remain a hurdle of the transhumanism movement.

Longevity pharmacologics have the potential to provide genetic optimization, potentially narrowing the gap between laboratory research and bedside application. Pharmacologic and biologic senolytics selectively clear senescent cells to decrease senescence-associated secretory phenotype (SASP) that cause organ function decline and aging [104]. Current clinical trials are underway, including dasatinib (kinase inhibitor) and quercetin (flavonoid plant pigment) for Alzheimer’s, childhood cancer survivors and idiopathic pulmonary fibrosis [104,105] and fisetin (a naturally occurring flavonoid compound) for osteoarthritis, frailty, and COVID-19 complications [106]. Additional pharmacologics (metformin, rapamycin), natural products (polyphenols, saponins, alkaloids, polysaccharides) [107], vitamins and minerals (nicotinamide riboside, vitamin C, E, Coenzyme Q10, collagen), and dietary supplements (epigallocatechin gallate in green tea, curcumin in turmeric, crocin in saffron, resveratrol found in red wine) have garnered attention for their potential to enhance the healthspan. In 2023, six chemical combinations were identified as inducing the Yamanaka factors (OSKM) and reversing transcriptomic age [108]. This marks a paradigm shift from previous work that suggested aging can be slowed chemically but can only be reversed genetically. While promising, the burden of proof has not yet been met for chemical longevity interventions, and traditional approaches such as smoking cessation [109], caloric restriction [55,110,111], intermittent and periodic fasting [112,113], stress reduction [114,115], and exercise [116,117] remain infallible.

## 4. Challenges and Ethical Considerations in the Pursuit of Immortality

The journey from anti-aging to immortality is currently awaiting the translation of animal study findings to humans, and progress is dependent on scientific clarity and humanity’s moral trajectory. Multi-disciplinary collaborations within the medical community are uniquely positioned to contribute across three critical areas bridging scientific investigations, clinical practice, and policy making [26,81,118].

The rapidly growing anti-aging market is valued at around USD 47 billion (2023) and expected to increase to USD 80 billion by 2030 [119]. Neuromodulator injections remained the most popular minimally invasive procedure in the United States in 2023 with nearly 9.5 million procedures performed (a 9% increase from the prior year), suggesting that the anti-aging market continues to flourish [120]. However, the globalization of anti-aging and life extension, in conjunction with current global overpopulation, raises profound ethical and philosophical questions, challenging our perceptions of life, death, and human existence. Contrary to popular belief, mathematical models have shown that even in the most radical immortality scenarios (no aging after 60 years old), overall population growth would remain relatively slow and would not result in catastrophic population consequences [121]. Instead, the primary limitations in advancing anti-aging and immortality remain related to scientific discovery, scalability, and the need for cultural and organizational adaptation.

A primary limitation of pursuing immortality comprises the technological risks, including unintended consequences, of immortality technologies. For example, gene editing may result in genetic mutations or unforeseen side effects, posing risks to human health and environmental stability, necessitating cautious development and regulation. Transhumanist ideologies, advocating for the transcendence of human limitations through technological augmentation, pose ethical dilemmas regarding equity and access. Disparate access to immortality interventions may exacerbate existing socioeconomic disparities, widening the gap between the privileged few who can afford longevity treatments and the marginalized majority. This pattern is already evident in both cosmetic and reconstructive plastic surgery, where the class divide is widened due to cost-prohibitive access to non-invasive and invasive esthetic procedures and where socioeconomic class is a predictor of reconstructive surgery outcomes [122]. Professional societies must address these ethical considerations while embracing innovation and responsible practice. This includes ensuring patient autonomy, informed consent, and equitable distribution of resources. Furthermore, the medical community has a unique opportunity to foster dialog and collaboration across disciplines, promoting ethical discourse and responsible stewardship in anti-aging technologies.

Heterochronic parabiosis is a sensational demonstration of science and society as the two driving forces within the field of immortality. While significant work is required to verify efficacy and safety, this emerging longevity frontier received a premature reveal in 2023, when Bryan Johnson, millionaire transhumanist and CEO of Project Blueprint, received a plasma transfusion from his 17-year-old son. Heterochronic parabiosis is an unprecedented intersection of plasma-based rejuvenation and allotransplantation [123]. The public looks to individuals in the medical community to provide expertise on safe and relevant technology developments in longevity. Social and popular media has played a key role in the re-emergence of “Homo evolutis” in popular culture, increasing exposure to the scientific community while propagating fallacies and disillusions. Plastic surgery is one of the most visible specialties on social media [124], providing the opportunity to collaborate with other medical specialties to educate the public, address misinformation, and advocate for patient needs [124,125,126]. Furthermore, as experts in esthetic enhancement, plastic surgeons can offer valuable insights into the psychosocial aspects of immortality aspirations, including the impact on body image and self-perception [125].

## 5. Beyond the Scalpel: Future Pathways of Plastic Surgery in Immortality

While advancements in medical technology continue to work towards physical immortality (avoidance of death) and biological immortality (absence of aging), the pursuit of immortality remains an elusive frontier, requiring adjunctive investigations of subjective immortality. Emerging technologies such as nanorobots, cryogenic sleep, therapeutic hypothermia, and cloning strategies hold potential to grant humans life beyond bodily death [127,128,129]. Artificial intelligence, mind cloning, and mind uploading once existed only as thought experiments of science fiction but are now an attainable possibility for virtual immortality [130,131].

Lifestyle and motivational counseling continue to be a crux of plastic surgery practice, as the synergy of lifestyle modifications with plastic surgery interventions improves patient outcomes and satisfaction. A record number of longevity biotechnology companies (LBCs), referred to as the “longevity boom” of 2023 [132], are now advertising endless services of lifestyle modifications, dietary supplementations, biological age testing and profiling, and longevity counseling. Individuals are now able to determine their “biologic age” through DNA methylation testing, identify aging risk factors, and implement potential lifestyle and habit modifications to decelerate biological aging [133,134]. However, the longevity boom poses substantial regulatory challenges; regulatory bodies such as the Food and Drug Administration (FDA) must use old statutes to address new drugs and devices, resulting in regulatory lag. For unregulated interventions, such as anti-wrinkle creams, the FDA can protect consumers from economic damage using the Misbranding Provision or threaten to re-classify the product as a drug subject to regulatory approval and threaten the manufacturer with seizure actions [135]. For protection against physical harm, the FDA is able to use the Adulteration Provision, which defines and prohibits goods that are contaminated, unsafe, or of poor quality. However, these provisions are often difficult, expensive, and slow. With limited resources, the FDA focuses only on the most pressing safety issues, incompletely addressing economic and physical injuries from anti-aging products [135]. Misleading claims and exaggerated promises surrounding immortality interventions can perpetuate unrealistic expectations, and subsequent physical and economic injuries may undermine public trust in scientific credibility, hindering genuine advancements in the field [136]. Alternatively, there has been increasing effort from scientific and medical communities to have the FDA classify aging as a disease, thus accelerating drug discovery and approval [137]. Thus, the medical community and professional societies should be prepared to address the sensationalism, educate the public, enhance public trust, support scientific endeavors, and foster informed discourse on the societal implications of immortality research to promote ethical engagement. Professional societies and academia have the power to hold new technologies accountable for physical and economic damages [135], advocate for responsible innovation, and provide evidence-based best practice guidelines.

In conclusion, the field of immortality and anti-aging is rapidly evolving and driven by advances in genetics, cellular biology, and regenerative medicine. The dichotomy of scientific conservatism and societal sensationalism now shapes the landscape of anti-aging medicine, including ethical considerations, regulatory challenges, and cultural attitudes towards aging, requiring scientists and surgeons to now become spokesmen. The medical community, particularly plastic surgeons, play a pivotal role in translating scientific discoveries into clinical practice, offering innovative solutions for rejuvenation and regeneration and closing the gap between lifespan and healthspan. Professional societies and academia should establish international multi-disciplinary collaborations and promote knowledge-sharing initiatives to accelerate progress and leverage diverse expertise and resources across borders.

## Figures and Tables

**Table 1:** Organisms exhibiting biological immortality or extreme longevity.

Organism (Scientific, Common Name)	Immortal Trait	Lifespan (Approx.)	Biological Mechanism
*Turritopsis dohrnii* (Immortal jellyfish)	Life-cycle reversal	Potentially indefinite	Cellular transdifferentiation allows medusa to revert back to polyp repeatedly [5]
	Rejuvenation		
*Hydra* spp. (Hydra)	Continuous tissue renewal	Indefinite (no senescence observed in lab)	Abundant stem cell self-renewal, maintained by FoxO transcription factor [6]; strong association with human FoxO3a gene in human longevity and maintenance of adult stem cells [7]
*Schmidtea mediterranea* (Planarian flatworm)	Regeneration/rejuvenation	Indefinite under lab conditions	Pluripotent neoblasts (adult stem cells) enable whole-body regeneration [8]
*Cheloniidae*/*Testudinidae* (Turtles, tortoises)	Negligible senescence	100–190+ years	Stable mortality rates and sustained physiological function with age [9]
*Arctica islandica* (Ocean quahog clam)	Extreme longevity	Up to 507 years	Low metabolic rate in cold marine environments; resistance to oxidative stress [10]
*Monorhaphis chuni* (Deep-sea sponge)	Extreme longevity	10,000+ years (est.)	Slow growth and metabolism in stable deep-sea environment [3]

**Table 2:** Plastic and reconstructive surgery investigations in immortality, regeneration, and rejuvenation.

Subspecialty	Subject/Examples	Explanation
Craniofacial	Gene editing for congenital anomalies (e.g., CRISPR for microtia, craniofacial syndromes)	Genetic editing offers opportunities to correct congenital malformations at their source, with potential extension to age-related craniofacial degeneration.
Hand and peripheral Nerve	Stem cell and pharmacologic therapies for peripheral nerve regeneration	Studies show improved regeneration of nerve gaps and functional recovery, offering insights into neural rejuvenation.
Microsurgery/reconstruction	Vascularized tissue engineering (flaps, composite grafts, bioengineered scaffolds)	Advances in engineered tissues with sustained vascularization demonstrate principles of long-term tissue replacement and regeneration.
	Tissue engineering of skin, auricular cartilage, trachea, and bone	Proof-of-concept constructs show how functional tissues can be biofabricated, supporting regeneration beyond natural lifespan.
	Stem cell-enhanced wound healing and radiation injury prevention	Plastic surgery models demonstrate how regenerative strategies overcome senescence and tissue damage, relevant to age-related degenerative processes.
Esthetic	Rejuvenation therapies (PRP, fat grafting, fillers with PDRN, PLLA, lasers, microneedling) for skin rejuvenation and alopecia	Serve as translational models of tissue renewal, collagen regeneration, and reversal of cellular senescence in the dermis.
Lymphedema	Stem cell-based and tissue-engineered lymphatic reconstructions	Illustrates the capacity to restore complex vascular/lymphatic systems, essential for maintaining tissue youthfulness.
Systemic/ transdisciplinary	Heterochronic parabiosis and young plasma studies	Though beyond conventional plastic surgery, these investigations overlap with reconstructive biology, focusing on systemic rejuvenation.

## Data Availability

Data sharing is not applicable.

## Abbreviations

The following abbreviations are used in this manuscript:

AI	Artificial intelligence
ARD	Age-related diseases
ATP	Adenosine triphosphate
Cas9	CRISPR-associated protein 9
COVID-19	Coronavirus disease 2019
CRISPR	Clustered Regularly Interspaced Short Palindromic Repeats
CSF	Cerebrospinal fluid
DNA	Deoxyribonucleic acid
FDA	U.S. Food and Drug Administration
FoxO	Forkhead box O
FoxO3a	Forkhead box O3A
IR	Infrared
IV	Intravenous
KLF4	Kruppel-like factor 4
LBCs	Longevity biotechnology companies
LLLT	Low-level laser (light) therapy
NIR	Near-infrared
nNIR	Near-infrared LED
OCT4	Octamer-binding transcription factor 4 (POU5F1)
OSK	OCT4–SOX2–KLF4
OSKM	OCT4–SOX2–KLF4–c-MYC
PDRN	Polydeoxyribonucleotide(s)
PLLA	Poly-L-lactic acid
PRP	Platelet-rich plasma
SASP	Senescence-associated secretory phenotype
SOX2	SRY-box transcription factor 2
c-MYC	MYC proto-oncogene
